# Homozygous *FIGLA* missense variant in two Japanese sisters with primary ovarian insufficiency: Case reports and literature review

**DOI:** 10.1002/rmb2.12635

**Published:** 2025-02-01

**Authors:** Wataru Tanikawa, Hirotomo Saitsu, Yasuhiko Nakamura, Yuichiro Shirafuta, Yasuko Fujisawa, Maki Fukami, Norihiro Sugino, Tsutomu Ogata

**Affiliations:** ^1^ Department of Pediatrics Hamamatsu University School of Medicine Hamamatsu Japan; ^2^ Department of Biochemistry Hamamatsu University School of Medicine Hamamatsu Japan; ^3^ Department of Obstetrics and Gynecology Yamaguchi Prefectural Grand Medical Center Hofu Japan; ^4^ Department of Obstetrics and Gynecology Yamaguchi University School of Medicine Ube Japan; ^5^ Department of Molecular Endocrinology National Research Institute for Child Health and Development Tokyo Japan; ^6^ Department of Pediatrics Hamamatsu Medical Center Hamamatsu Japan

**Keywords:** disease‐causing, *FIGLA*, inheritance, primary ovarian insufficiency, whole genome sequencing

## Abstract

**Background:**

*FIGLA* is a transcription factor gene which plays a critical role in folliculogenesis. Consistent with this, *FIGLA* variants have been identified in females with non‐syndromic primary ovarian insufficiency (POI) in both autosomal‐dominant and autosomal‐recessive forms.

**Case Description:**

We encountered two Japanese sisters who had secondary or primary amenorrhea at 15 years of age. They were diagnosed as having non‐syndromic primary ovarian insufficiency (POI) with hypergonadotropic hypoestrogenism and markedly low serum anti‐Müllerian hormone values.

**Outcome:**

Whole genome sequencing revealed a novel homozygous missense variant, NM_001004311.3:c.338A>G:p.(Tyr113Cys), in *FIGLA* essential for folliculogenesis in the two sisters. The parents were heterozygous for this variant, and the heterozygous mother had regular menses at 51 years of age. This variant was extremely rare in public databases, and was invariably assessed as deleterious by six prediction tools. Furthermore, the p.(Tyr113Cys)‐FIGLA protein was assessed as “pathogenic” or “likely pathogenic” by protein structural predictions, and was evaluated as “destabilizing” or “decrease stability” by protein stability predictions

**Conclusion:**

The results, in conjunction with the data reported in the literature, imply that *FIGLA* variants account for a small but certain fraction of non‐syndromic POI, and pose a question as to the relevance of *FIGLA* variants to an autosomal dominant form of POI, although *FIGLA* variants have been identified in both autosomal dominant and autosomal recessive forms of non‐syndromic POI.

## Graphical abstract

FIGLA is a transcription factor gene which is expressed in the very early stage of female germ cells and plays a critical role in folliculogenesis. Here, we report a homozygous FIGLA variant identified in two Japanese sisters with non‐syndromic primary ovarian insufficiency (POI), and review the previously reported autosomal‐dominant and autosomal‐recessive FIGLA variants. The results imply that FIGLA variants account for a small but certain fraction of non‐syndromic POI and pose a question as to the presence of autosomal‐dominant FIGLA variants.

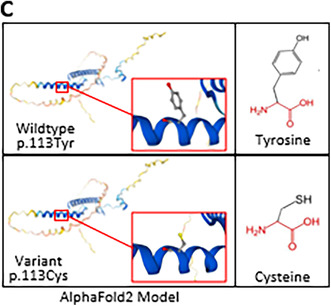

## 1 INTRODUCTION

Primary ovarian insufficiency (POI) is a rare reproductive disorder characterized by primary amenorrhea (PA) or secondary amenorrhea (SA) with hypergonadotropic (FSH ≥40 IU/L) hypoestrogenism in women below 40 years of age.^1^, ^2^ It takes place as a non‐syndromic form without extragenital features or as a syndromic form with extragenital features.^1^, ^2^ POI is a heterogeneous condition caused by various factors including autoimmune, chromosomal, and gene abnormalities.^1^, ^2^ Although underlying factors still remain elusive in many females with POI, a number of causative genes have been revealed by next generation sequencing.^2^

FIGLA (factor in germline alpha or folliculogenesis specific basic helix–loop–helix (bHLH) transcription factor) on chromosome 2p13.3 is a transcription factor gene containing the bHLH domain which typically binds to a consensus DNA sequence (CANNTG) called an E‐box and transactivates target genes.^3^ *FIGLA* is primarily expressed in the very early stage of female germ cells,^4^ and regulates the expression of zona pellucida genes (*ZP1*, *ZP2*, and *ZP3*) and multiple oocyte‐specific genes.^5^ *Figla* knockout female mice are infertile because of folliculogenic failure, whereas *Figla* knockout male mice are fertile.^6^ Consistent with these findings, several rare pathogenic or likely pathogenic variants have been identified in *FIGLA* of patients with non‐syndromic POI (OMIM #612310: premature ovarian failure 6), as well as several rare variants of unknown significance (VUS).^7^, ^8^, ^9^, ^10^, ^11^, ^12^, ^13^, ^14^, ^15^ Notably, such variants are present in both a homozygous condition consistent with an autosomal‐recessive inheritance and a heterozygous condition indicative of an autosomal‐dominant inheritance.^7^, ^8^, ^9^, ^10^, ^11^, ^12^, ^13^, ^14^, ^15^

Here, we report a homozygous *FIGLA* variant identified in two Japanese sisters with non‐syndromic POI, and review the previously reported *FIGLA* variants. The results imply that *FIGLA* variants account for a small but certain fraction of non‐syndromic POI, and argue against the presence of autosomal‐dominant POI.

## 2 CASE REPORTS

We encountered two Japanese sisters with POI. The elder sister was referred to Yamaguchi Prefectural Grand Medical Center (a branch hospital of Yamaguchi University School of Medicine) at 15 5/12 years of age because of SA which occurred after three times of vaginal bleedings at 14 0/12, 14 4/12, and 14 5/12 years old. Three years later, the younger sister came to the same Center at 15 4/12 years of age because of PA.

The two sisters showed similar clinical findings at the time of first examination (Table 1). They exhibited normal body size, poor secondary sexual development, hypergonadotropic hypoestrogenism with markedly low serum anti‐Müllerian hormone (AMH) values, and normal or normal variant female karyotype. In addition, magnetic resonance imaging failed to identify ovary and revealed hypoplastic uterus in the elder sister. There was no extragenital abnormality in the two sisters. Thus, they were diagnosed with non‐syndromic POI, and were placed on a cyclic estrogen and progesterone replacement therapy which successfully induced secondary sex development and regular menses. On the latest examination, the 24‐year‐old elder sister measured 163 cm (+0.9 SD) and weighed 50 kg (−0.4 SD), and the 21‐year‐old younger sister measured 163 cm (+0.9 SD) and weighed 55 kg (+0.2 SD).

**TABLE 1:** Clinical findings of the two sisters with POI.

	Elder sister	Younger sister
Age at examination (y:m)	15:5	15:4
Height (cm, SDS)	155 (−0.4)	157 (±0)
Weight (kg, SDS)	50 (−0.2)	46 (−0.7)
Pubertal development		
Breast	Tanner 2	Tanner 1–2
Pubic hair	Positive^a^	Positive^a^
Ovary	Undetected on MRI	Not examined
Uterus	Hypoplastic on MRI	Not examined
Endocrine findings		
LH (mIU/mL)	21.4	30.5
FSH (mIU/mL)	75.3	96.9
Estradiol (pg/mL)	<10	<10
AMH (ng/mL)	0.02	0.02
Cytogenetic examination		
Karyotype	46,XX,inv(9)(p12q13)	46,XX

*Note*: Age‐ and sex‐matched Japanese reference values: LH, 0.2–44.4; FSH 1.6–10.6; estradiol, 10–250; and AMH, 0.8–14.2. Conversion factor to SI unit: 1.0 for LH and FSH (IU/L), 3.67 for estradiol (pmol/L), and 7.14 for AMH (pmol/L).

Abbreviations: AMH, anti‐Müllerian hormone; FSH, follicle stimulating hormone; LH, luteinizing hormone; m, month; MRI, magnetic resonance imaging; y, year.

^a^ According to the patients and the mother.

The 51‐year‐old mother and the 60‐year‐old father were clinically normal, and the mother had regular menses (the average menopausal age in Japanese females, ~50 years) (https://www.jsog.or.jp/citizen/5717/). Allegedly, they were derived from the same district and had a distant common relative, although a precise pedigree was unknown. There was no subject with POI in the paternal and maternal relatives.

## 3 MOLECULAR STUDIES

We performed whole genome sequencing (WGS) using leukocyte genomic DNA samples of the sisters and their parents. This study was approved by the Institutional Review Board Committees at Hamamatsu University School of Medicine and Yamaguchi University School of Medicine, and was performed after obtaining written informed consent. WGS libraries were generated by using MGIEasy PCR‐free DNA Library Prep set (MGI). Sequencing was performed on a DNBSEQ‐G400 (MGI) in a paired‐end mode (2x 151 nt). Sequenced reads were aligned to the GRCh38 reference genome using Clara Parabricks v4.2.0‐1 fq2bam (NVIDIA, Santa Clara, CA) and variant calling was performed using deepvariant (NVIDIA). Annotation and variant filtering were performed as described previously.^16^

**FIGURE 1:**
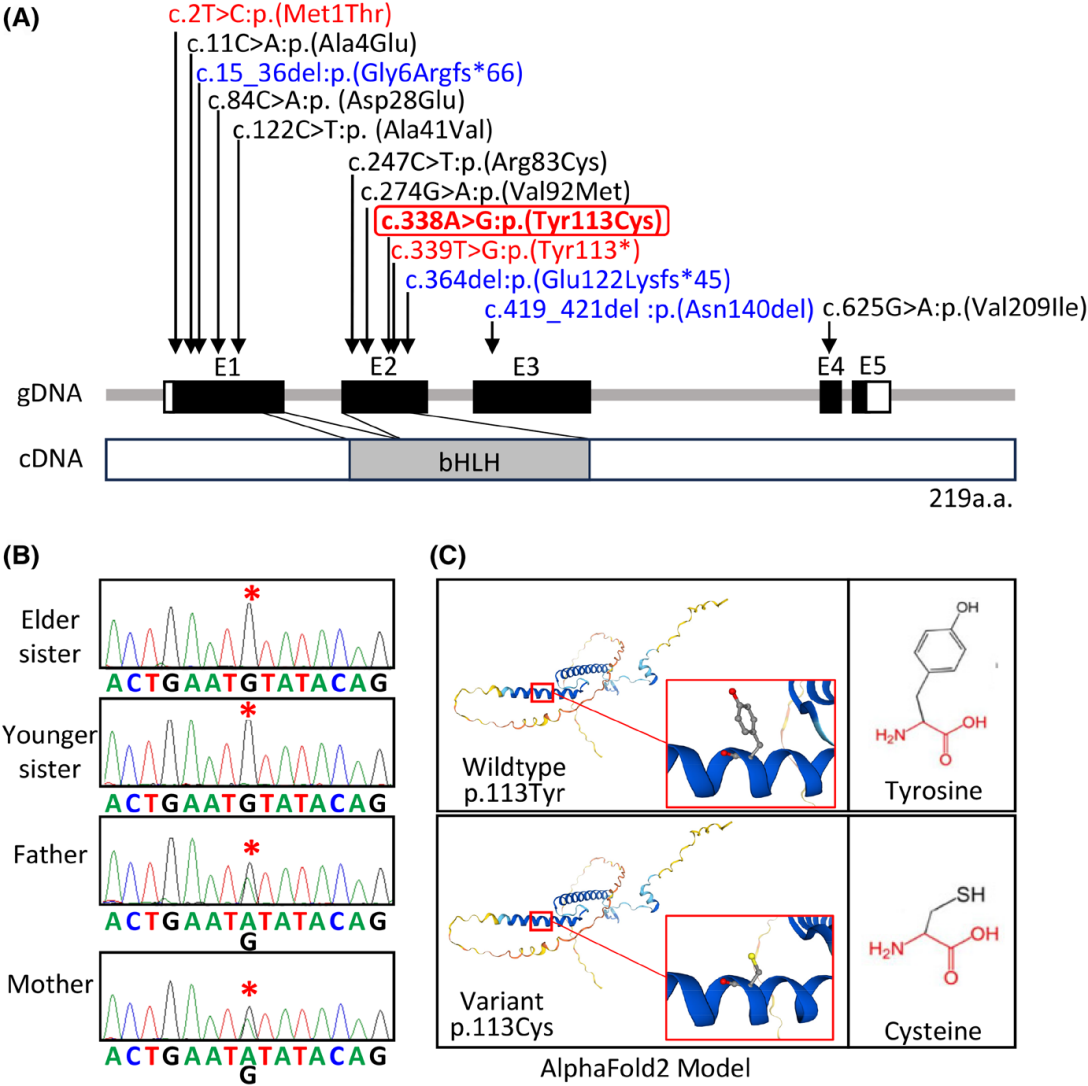
*FIGLA* variant identified in this study. (A) The gDNA and cDNA structures of *FIGLA* and the positions of the c.338A>G:p.(Tyr113Cys) found in this study and the 11 variants reported to date. Homozygous variants 1–3 are shown in red (c.338A>G:p.(Tyr113Cys) is surrounded with a rectangle), heterozygous variants 4–6 (which have been assessed as pathogenic or likely pathogenic) in blue, and heterozygous variants 7–12 (which have been evaluated as VUS) in black. The c.338A>G:p.(Tyr113Cys) variant resides on exon 2 encoding the bHLH domain spanning from ~65 to ~123 amino acids. (B) Electrochromatograms showing the c.338A>G substitution present in a homozygous condition in the two sisters and in a heterozygous condition in the parents (indicated with asterisks). The primers utilized are as follows: Forward, 5′‐ctcagacaaggaggcagtgg‐3′; and reverse, 5′‐tcacttggcacagtgtctcg‐3′. (C) Wild‐type and variant FIGLA proteins predicted by AlphaFold2. The backbone common to Tyrosine and Cysteine is colored red, and the side chain unique to each amino acid is colored black.

Consequently, we identified a novel homozygous *FIGLA* missense variant, NM_001004311.3:c.338A>G:p.(Tyr113Cys), in exon 2 encoding the bHLH domain in the two sisters (Figure 1A). This variant was confirmed by Sanger sequencing (Figure 1B). The parents were heterozygous for this variant. This variant was extremely rare in the public databases utilized in this study, and was predicted as deleterious by all the six in silico prediction tools utilized in this study (Table 2). No other rare variant with a frequency of ≤0.005 was detected in POI‐related genes including those listed in the following references.^1^, ^2^, ^13^, ^15^

**TABLE 2:** Assessment of *FIGLA* c.338A>G:p.(Tyr113Cys).

Frequency in public databases	
gnomAD v4.1.0_exome_EAS	0.00002238
HGVD	0.000413
54KJPN	0.000018
Pathogenicity predictions	
CADD GRCh38‐v1.6 (PHRED score)	1% Most deleterious (26.3)
Polyphen‐2 HumVar (score)	Possibly damaging (0.452)
SIFT (score)	Damaging (0.00)
MutationTaster2021 (Tree vote: del | benign)	Deleterious (74|26)
BayesDel (no allele frequency) (score)	Pathogenic strong (0.5372)
REVEL (score)	Pathogenic moderate (0.899)
Protein structural predictions	
EVE (score)	Pathogenic (0.729)
AlphaMissense (score)	Likely pathogenic (0.975)
Protein stability predictions	
DynaMut (ΔΔG kcal/mol)	Destabilizing (−1.038)
MUpro (ΔΔG kcal/mol)	Decrease stability (−0.552)

The URLs utilized in the assessment are as follows:

gnomAD (Genome Aggregation Database): http://gnomad.broadinstitute.org.

HGVD (Human Genetic Variation Database): http://www.hgvd.genome.med.kyoto‐u.ac.jp;

54KJPN (Whole‐genome sequences of approximately 54 000 healthy Japanese individuals and construction of the highly accurate Japanese population reference panel): https://jmorp.megabank.tohoku.ac.jp.

CADD (Combined Annotation–Dependent Depletion): http://cadd.gs.washington.edu/score; PHRED scores of >10 are regarded as 10% most deleterious, and those of >20 indicates the 1% most deleterious.

Polyphen‐2 HumVar: http://genetics.bwh.harvard.edu/pph2; Scores range from 0.000 (most probably benign) to 1.000 (most probably damaging).

SIFT (Sorting Intolerant From Tolerant): http://sift.jcvi.org; Scores of ≤0.05 and those >0.05 are assessed as damaging and tolerated, respectively.

MutationTaster2021 (GRCh37/Ensembl 102): https://www.genecascade.org/MutationTaster2021; The Tree vote numbers, denoted as deleterious|benign, indicate the confidence of the classifier that the variant belongs to that particular class.

BayesDel: https://fenglab.chpc.utah.edu/BayesDel.html; Scores range from −1.29334 (most benign) to 0.75731 (most pathogenic).

REVEL (Rare Exome Variant Ensemble Learner): https://sites.google.com/site/revelgenomics; Scores range from 0.000 (most benign) to 1.000 (most pathogenic).

EVE (evolutionary model of variant effect): https://evemodel.org; Scores range from 0.000 (most benign) to 1.000 (most pathogenic).

AlphaMissense: https://alphamissense.hegelab.org; Scores range from 0.000 to 1.000, and are interpreted as the approximate probability of a variant being clinically pathogenic.

DynaMut: http://biosig.unimelb.edu.au/dynamut; ΔΔG (kcal/mol) represents the prediction change in Gibbs free energy, and lower than 0 indicates destabilizing.

MUpro: http://mupro.proteomics.ics.uci.edu; ΔΔG (kcal/mol) indicates the prediction of the change in Gibbs free energy using support vector machines and regression methods, and lower than 0 indicates decreased stability.

We also examined the consanguinity of the parents. While PLINK analysis did not support consanguinity between the parents (Table S1), application of AutoMap and H3M2, which are utilized to find regions of homozygosity or runs of homozygosity (ROH) from WGS data, revealed that the two sisters had several ROH regions including a chromosome 2p13.3 segment involving *FIGLA* (Figure S1). This indicated that the parents were distally related and shared several genomic segments in common.

## 4 PROTEIN STRUCTURE AND STABILITY PREDICTIONS

We examined the p.Tyr113Cys‐FIGLA protein using several in silico tools (Table 2). First, since FIGLA protein has not been registered in Protein Data Bank, we predicted the 3D‐structure of the wild‐type protein with Tyr113 residue and the variant protein with Cys113 residue using AlphaFold2 (https://alphafold.ebi.ac.uk/) (Figure 1C). The Cys residue was smaller and more hydrophobic than the Tyr residue, and it was predicted that the smaller size may lead to loss of interactions with other molecules, and that the hydrophobic property may result in loss of hydrogen bonds and/or disturb correct folding. The structure of the predicted variant protein with Cys113 residue was assessed as “pathogenic” by EVE and “likely pathogenic” by AlphaMissense. Furthermore, the predicted variant protein was evaluated as “destabilizing” by DynaMut and “decrease Stability” by MUpro.

## 5 DISCUSSION

This study revealed a homozygous *FIGLA* missense variant, c.338A>G:p.(Tyr113Cys), in the two sisters with non‐syndromic POI. In this regard, we performed WGS rather than whole exome sequencing (WES), to examine various underlying factors including sequence, copy number, and structural variants in both coding and noncoding regions. In particular, since genetic origin of POI with an autosomal‐recessive inheritance as well as a female‐limited autosomal dominant inheritance is postulated in this family, we attempted not to overlook a deleterious aberration in noncoding regions such as an intronic variant affecting splicing in one allele of a POI‐related gene in which a deleterious exonic variant is identified in another allele. In addition, WGS permits more precise assessment for consanguinity than WES, as employed in this study.

The c.338A>G:p.(Tyr113Cys) variant is assessed as VUS by the ACMG guideline^17^ which is continuously revised and updated after the first publication in 2015 (ClinGen Sequence Variant Interpretation Working Group, https://clinicalgenome.org/working‐groups/sequence‐variant‐interpretation/), because it is positive for three categories (PM1, PM2‐supporting, and PP3‐strong) (for the explanation of categories in the ACMG assessment, see footnotes in Table 3). It is notable, however, that (1) this variant is extremely rare in the general population and is predicted to have high pathogenicity by all the six in silico tools; (2) the POI phenotype with extremely low AMH values indicative of the folliculogenic failure before the development of AMH‐producing preantral and small antral follicles^18^, ^19^ is consistent with the defective function of *FIGLA* which is expressed in a very early stage of female germ cells and is required for folliculogenesis^4^, ^5^, ^6^; (3) no other rare variant was revealed in POI‐related genes listed in several papers^1^, ^2^, ^13^, ^15^; and (4) the protein structure and stability predictions argue for the pathogenic loss‐of‐function effect of this variant. Collectively, although functional studies were not performed for the variant p.Tyr113Cys‐FIGLA protein, it is likely that this variant is the primary disease‐causing factor for the development of non‐syndromic POI in the two sisters.

**TABLE 3:** Summary of *FIGLA* variants identified in non‐syndromic POI.

**Variant**	Variant 1	Variant 2	Variant 3	Variant 4	Variant 5	Variant 6	Variant 7	Variant 8	Variant 9	Variant 10	Variant 11	Variant 12
NM_001004311.3	c.338A>G	c.2T>C	c.339T>G	c.15_36del	c.364del (1)	c.419_421del (2)	c.11C>A	c.84C>A	c.122C>T	c.625G>A	c.247C>T (3)	c.274G>A (4)
NP_001004311.2	p.(Tyr113Cys)	p.(Met1Thr)	p.(Tyr113Ter)	p.(Gly6ArgfsTer66)	p.(Glu122LysfsTer45)	p.(Asn140del)	p.(Ala4Glu)	p.(Asp28Glu)	p.(Ala41Val)	p.(Val209Ile)	p.(Arg83Cys)	p.(Val92Met)
dbSNP	rs1675980463	rs1001164504	Not registered	rs587776535	rs1553390227	rs71647804	rs71647803	rs373561603	rs1210411819	rs186548772	rs369140654	rs1304093445
Zygosity	Homozygous	Homozygous	Homozygous	Heterozygous	Heterozygous	Heterozygous	Heterozygous	Heterozygous	Heterozygous	Heterozygous	Heterozygous	Heterozygous
Inheritance	AR	AR	AR	AD	AD	AD	AD	AD	AD	AD	AD	AD
Transmission	Biparental (Distally related)	Biparental (Related)	Probably biparental (Related) (5)	Paternal	Unknown	Unknown	Unknown (*n* = 1) Paternal (*n* = 2)	Paternal	Unknown	Paternal	Unknown	Unknown
**Patient**	Patients 1–2 (6)	Patients 3–6 (7)	Patient 7 (8)	Patient 8	Patient 9	Patient 10	Patients 11–13 (9)	Patient 14	Patient 15	Patient16	Patient 17	Patient 18
Ethnicity	Japanese	Chinese	Turkish	Chinese	Italian	Chinese	Chinese	Chinese	French	Chinese	Indian	Unknown
PA or SA	SA and PA	PA	Probable PA	SA	Not described	SA	Not described	Not described	Not described	Not described	Not described	Neither (SMD)
Note	Fertility in a heterozygous mother	Fertility in heterozygous mothers	Probable fertility in a heterozygous mother (10)									
**Frequency in gnomAD (v4.1.0)**												
Both sexes	0.00002238 (East Asian)	0.000 (East Asian)	0.000 (Total)	0.000 (East Asian)	0.0000008621 (European)	0.000 (East Asian)	0.0004459 (East Asian)	0.001579 (East Asian)	0.00008462 (European)	0.003242 (East Asian)	0.00001106 (South Asian)	0.000004960 (Total)
Female	0.000	0.000	0.000	0.000	0.000001654	0.000	0.0006318	0.001624	0.00008509	0.003173	0.00004327	0.000006157
Male	0.00004581	0.000	0.000	0.000	0.000	0.000	0.0002465	0.001531	0.00008410	0.003313	0.000	0.000003746
Homozygote	0.000	0.000	0.000	0.000	0.000	0.000	0.000	0.000025 (male)	0.000	0.000	0.000	0.000
**In silico pathogenicity predictions**												
CADD (GRCh38‐v1.6) (Phred score)	1% Most deleterious (26.3)	1% Most deleterious (22.6)	1% Most deleterious (36.0)	1% Most deleterious (23.7)	1% Most deleterious (28.0)	Non‐deleterious (7.50)	10% Most deleterious (17.6)	Non‐deleterious (8.55)	1% Most deleterious (24.8)	10% Most deleterious (19.15)	1% Most deleterious (28.5)	1% Most deleterious (26.7)
PP2_HVAR (Score)	Possibly damaging (0.452)	Benign (0.108)	Not predicted^a^	Not predicted^a^	Not predicted^a^	Not predicted^a^	Benign (0.232)	Benign (0.02)	Benign (0.418)	Benign (0.055)	Probably damaging (0.939)	Probably damaging (0.998)
SIFT (Score)	Damaging (0.00)	Damaging (0.00)	Not predicted^a^	Not predicted^a^	Not predicted^a^	Not predicted^a^	Damaging (0.005)	Benign Moderate (0.361)	Benign Moderate (0.098)	Benign Moderate (0.196)	Damaging (0.014)	Damaging (0.001)
MutationTaster2021 (Tree vote: del|benign)	Deleterious (74|26)	Deleterious (140|60)	Deleterious (188|12)	Not predicted^a^	Not predicted^a^	Not predicted^a^	Benign (6|94)	Benign (5|95)	Benign (17|83)	Benign (2|98)	Benign (6|94)	Benign (28|72)
BayesDel (no allele frequency) (Score)	Pathogenic strong (0.5372)	Pathogenic strong (0.6299)	Pathogenic strong (0.6299)	Not predicted^a^	Not predicted^a^	Not predicted^a^	Benign supporting (−0.1173)	Benign strong (−0.4197)	Benign supporting (−0.0504)	Benign moderate (−0.3018)	Uncertain (0.04511)	Pathogenic moderate (0.4296)
REVEL (Score)	Pathogenic moderate (0.899)	Uncertain (0.578)	Not predicted^a^	Not predicted^a^	Not predicted^a^	Not predicted^a^	Benign moderate (0.287)	Benign moderate (0.312)	Uncertain (0.528)	Benign moderate (0.155)	Uncertain (0.599)	Pathogenic supporting (0.780)
**ACMG assessment** (Category)	VUS (11) (PM1, PM2‐supporting, PP3‐strong)	Likely Pathogenic (PVS1‐moderate, PS3, PM2‐supporting)	Likely pathogenic (PVS1, PM2‐supporting)	Likely pathogenic (PVS1, PM2‐supporting)	Likely pathogenic (PVS1, PM2‐supporting)	Likely pathogenic (PS3, PM2‐ supporting, PM4)	VUS (PS3)	VUS (PS3)	VUS (PS3, PM2‐ supporting)	VUS (PS3)	VUS (PM1, PM2‐ supporting)	VUS (PM1, PM2‐supporting, PP3‐ moderate)
**ClinVar evaluation**	Not registered	Pathogenic	Not registered	Pathogenic	Not registered	Pathogenic	VUS	Benign	Not registered	Benign	VUS	Not registered
**Remark**	Footnote‐1	Footnote‐2	Footnote‐3	Footnote‐3	Footnote‐3	Footnote‐4	Footnote‐5	Footnote‐6	Footnote‐7	Footnote‐6		
**References**	This study	[^10^, ^11^]	[^15^]	[^7^]	[^12^]	[^7^]	[^7^, ^14^]	[^14^]	[^9^]	[^14^]	[^8^]	[^13^]

Abbreviations: AD, autosomal dominant; AR, autosomal recessive; PA, primary amenorrhea; SA, secondary amenorrhea; SMD, secondary menstrual disturbance; VUS, variant of uncertain significance.

^a^ Pathogenic prediction is frequently impossible for nonsense, frameshift, and insertion/deletion variants.

(1) Coexistence with a heterozygous *NOBOX* variant (NM_001080413.3:c.1626del:p(Phe543SerfsTer7)) and a heterozygous *NR5A1* variant (NM_004959.5:c.1063G>A:p.(Val355Met)).

(2) Because of two consecutive Asn codons, this variant is also described as c.416_418del: p.Asn139del.

(3) Described as c.252C>T:p(Arg83Cys) in the original paper.

(4) Co‐existence with a heterozygous *NOBOX* variant (NM_001080413.3:c.131G>T:p(p.Arg44Leu)) and a heterozygous *DMC1* variant (NM_007068.3: c.598A>G:(p.Met200Val)).

(5) Probable biparental origin because of the consanguinity of parents (first‐degree cousin).

(6) Two sisters described in this report.

(7) Two sisters described by Chen et al.^10^ and two sisters described by Yuan et al..^11^

(8) The younger sister exhibits similar phenotype.

(9) One patient described by Zhao et al.^7^ and two unrelated patients reported by Mei et al..^14^

(10) Probable heterozygosity in the parents because of the consanguinity.

(11) This variant is considered to be disease‐causing (see text).

**ACMG assessment (category)**

PVS1: Null variant (nonsense, frameshift, canonical ±1 or 2 splice sites, initiation codon, single or multiexon deletion). PVS1 strength is classified as PVS1‐supporting, moderate, strong, or PVS1 according to the PVS1 decision tree described in Recommendations for interpreting the loss of function PVS1 ACMG/AMP variant criterion.

PS3: Well‐established in vitro or in vivo functional studies supportive of a damaging effect.

PM1: Presence on the well‐established functional domain.

PM2‐supporting: Absent from controls, or at extremely low frequency if recessive, in Exome Sequencing Project, 1000Genomes Project, or Exome Aggregation Consortium.

PM4: Protein length changes as a result of in‐frame deletions/insertions in a nonrepeat region or stop‐loss variants.

PP3: Pathogenicity determination result using a computational tool that reach at least strong evidence for pathogenicity and moderate for benignity.

**Remark**

Footnote‐1: This variant is predicted to have loss‐of‐function by the protein structure and stability predictions.

Footnote‐2: This variant affects the translation initiation codon, and the Met191 may have been utilized as an alternative translation start codon, producing a drastically short protein missing the bHLH domain.

Footnote‐3: These variants are predicted to undergo nonsense‐mediated mRNA decay.

Footnote‐4: This variant impairs the heterodimer formation with TCF3.

Footnote‐5: This variant permits the heterodimer formation with TCF3, but impairs transcriptional activity and binding activity for ZP1, ZP2, and ZP3.

Footnote‐6: These variants have reduced transcriptional activity and binding activity for ZP1, ZP2, and ZP3.

Footnote‐7: This variant disrupts the transcriptional activity of the E‐box containing promoter.

Several matters are notable in this family. First, the elder sister experienced three times of vaginal bleedings. This may imply that the variant retains some residual function which, together with the elevated gonadotropin stimulation, permitted a certain degree of folliculogenesis. Second, the heterozygous mother was fertile and showed regular menses at 51 years old. This argues for the recessive effect of this variant. Lastly, the parents were heterozygotes for the extremely rare variant. This would be consistent with a certain degree of parental consanguinity, as indicated by the presence of a relative common to the parents and the AutoMap and H3M2 data.

To the best of our knowledge, 11 rare *FIGLA* variants with frequencies of ≤0.005 have been identified in females with non‐syndromic POI (variants 2–12 in Table 3)^7^, ^8^, ^9^, ^10^, ^11^, ^12^, ^13^, ^14^, ^15^ (we updated relevant information of the variants, such as the frequencies in gnomAD database and in silico pathogenicity prediction data). In this regard, several findings are notable. First, the variants include apparently amorphic variants (variant 2 affecting the translation start codon, and nonsense variant 3 and frameshift variants 4 and 5 which satisfy the condition for the occurrence of nonsense‐mediated mRNA decay^20^), together with an in‐frame 3 bp deletion variant 6 and missense variants 7–12. Second, variants 2–4 and 6 are absent from the general female population and variant 5 is present in an extremely low frequency in the general female population, whereas variants 7–12 have been detected in the general female population with low but certain frequencies. Third, variants 2 and 7–12 are variably evaluated by in silico pathogenicity predictions (pathogenic prediction is frequently impossible for nonsense, frameshift, and insertion/deletion variants such as variants 3–6). Fourth, various studies have implicated some degrees of hypofunction, not necessarily definitive disease‐causing hypofunction, for variants 2–10 (see footnotes in the legend for Table 3). Lastly, variant 5 is associated with POI‐related *NOBOX* and *NR5A1* variants, and variant 12 is accompanied by POI‐related *NOBOX* and *DMC1* variants. Thus, although the data are complicated, variants 2–6 are assessed as “likely pathogenic,” and variants 7–12 are evaluated as “VUS,” on the basis of the revised and updated ACMG guideline^17^ (ClinGen Sequence Variant Interpretation Working Group, https://clinicalgenome.org/working‐groups/sequence‐variant‐interpretation/) (Table 3). This assessment is well consistent with the evaluation reported in ClinVar (https://www.ncbi.nlm.nih.gov). In this regard, the extremely low frequency of variant 5 in the general female population may be explained by assuming incomplete penetrance or the examination before the occurrence of SA. In addition, further two likely pathogenic variants in *FIGLA*, that is, c.326G>A:p.(Gly109Asp) and c.319C>T:p.(Leu107Phe), have been registered as the underlying factor for POI in ClinVar without detailed clinical findings.

Notably, variants 1–3 are present in a homozygous condition in POI females, whereas the remaining variants 4–12 are present in a heterozygous condition in POI females. In this context, it is unlikely that the difference in the inheritance pattern (autosomal recessive or autosomal dominant) is primarily due to the variation in the residual function, because variants 2 and 3 are regarded as apparently amorphic as are variants 4 and 5. Similarly, it is also unlikely that missense variants 6–12 have a dominant‐negative effect, because FIGLA protein forms a heterodimer with bHLH protein TCF3 rather than a homodimer.^7^

Thus, *FIGLA* variants may cause both autosomal‐recessive and autosomal‐dominant forms of POI, primarily depending on the condition of multiple other genetic and environmental factors. In this regard, homozygosity for variants 1–3 is associated with PA and SA, whereas heterozygosity for variants 4 and 6 is accompanied by SA (in addition, the VUS variant 12 is associated with secondary menstrual disturbance). This may suggest that homozygous *FIGLA* variants cause more severe POI phenotype than heterozygous *FIGLA* variants. However, this notion would remain speculative, because POI phenotype (PA or SA) has not been described for variants 5 and 7–11.

Alternatively, most, if not all, variants may not cause POI in a heterozygous condition. Indeed, Shekari et al. have recently found nearly all heterozygous variants involving protein‐truncating variants in POI‐related genes including *FIGLA* of women with normal reproductive function in UK biobank data, thereby claiming that such variants are regarded as either partially penetrant or not pathogenic and are unlikely to cause POI in a heterozygous condition.^21^ Consistent with this, apparently amorphic variants 2 and 3 have permitted normal ovarian function in the heterozygous mothers. In addition, while variants 4, 8, and 10 are transmitted from the father, variant 7 is of paternal or parent‐unknown origin and variants 5, 6, 9, 11, and 12 are unknown for parental origin. Thus, some of heterozygous variants may be derived from the fertile mothers. These findings would argue against the relevance of variants 4–6, which are assessed as pathogenic or likely pathogenic, as well as variants 7–12, which are present in low but certain frequencies in the normal population and are associated with low pathogenicity, to the development of POI in a heterozygous condition.

In summary, we identified a *FIGLA* missense variant which caused non‐syndromic POI in two homozygous sisters and permitted normal reproductive function in their heterozygous mother, and reviewed the updated data of 11 *FIGLA* variants reported to date. The results imply that *FIGLA* variants account for a small but certain fraction of non‐syndromic POI, and raise a question as to the presence of autosomal‐dominant POI.

## FUNDING INFORMATION

This work was supported by the Japan Agency for Medical Research and Development (AMED) (JP24ek0109760).

## CONFLICT OF INTEREST STATEMENT

The authors declare no conflict of interest.

## ETHICS STATEMENT

This study was approved by the Institutional Review Board Committees at Hamamatsu University School of Medicine and Yamaguchi University School of Medicine, and was performed after obtaining written informed consent.

## Supporting information


Data S1.

## Data Availability

The data related to this report are available upon reasonable request.

## References

[1] Tucker EJ , Grover SR , Bachelot A , Touraine P , Sinclair AH . Premature ovarian insufficiency: new perspectives on genetic cause and phenotypic spectrum. Endocr Rev. 2016;37(6):609–635. 27690531 10.1210/er.2016-1047

[2] França MM , Mendonca BB . Genetics of primary ovarian insufficiency in the next‐generation sequencing era. J Endocr Soc. 2019;4(2):bvz037. 32099950 10.1210/jendso/bvz037 PMC7033037

[3] Littlewood TD , Evan GI . Transcription factors 2: helix–loop–helix. Protein Profile. 1995;2(6):621–702. 7553065

[4] Huntriss J , Gosden R , Hinkins M , Oliver B , Miller D , Rutherfold AJ , et al. Isolation, characterization and expression of the human Factor In the Germline alpha (FIGLA) gene in ovarian follicles and oocytes. Mol Hum Reprod. 2002;8(12):1087–1095. 12468641 10.1093/molehr/8.12.1087

[5] Liang L , Soyal SM , Dean J . FIGalpha, a germ cell specific transcription factor involved in the coordinate expression of the zona pellucida genes. Development. 1997;124(24):4939–4947. 9362457 10.1242/dev.124.24.4939

[6] Soyal SM , Amleh A , Dean J . FIGalpha, a germ cell‐specific transcription factor required for ovarian follicle formation. Development. 2000;127(21):4645–4654. 11023867 10.1242/dev.127.21.4645

[7] Zhao H , Chen ZJ , Qin Y , Shi Y , Wang S , Choi Y , et al. Transcription factor FIGLA is mutated in patients with premature ovarian failure. Am J Hum Genet. 2008;82(6):1342–1348. 18499083 10.1016/j.ajhg.2008.04.018 PMC2427265

[8] Tosh D , Rani HS , Murty US , Deenadayal A , Grover P . Mutational analysis of the FIGLA gene in women with idiopathic premature ovarian failure. Menopause. 2015;22(5):520–526. 25314148 10.1097/GME.0000000000000340

[9] Bouilly J , Beau I , Barraud S , Bernard V , Azibi K , Fagart J , et al. Identification of multiple gene mutations accounts for a new genetic architecture of primary ovarian insufficiency. J Clin Endocrinol Metab. 2016;101(12):4541–4550. 27603904 10.1210/jc.2016-2152

[10] Chen B , Li L , Wang J , Li T , Pan H , Liu B , et al. Consanguineous familial study revealed biallelic FIGLA mutation associated with premature ovarian insufficiency. J Ovarian Res. 2018;11(1):48. 29914564 10.1186/s13048-018-0413-0 PMC6006558

[11] Yuan P , He Z , Sun S , Li Y , Wang W , Liang X , et al. Bi‐allelic recessive loss‐of‐function mutations in FIGLA cause premature ovarian insufficiency with short stature. Clin Genet. 2019;95(3):409–414. 30474133 10.1111/cge.13486

[12] Cattoni A , Spano A , Tulone A , Boneschi A , Masera N , Maitz S , et al. The potential synergic effect of a complex pattern of multiple inherited genetic variants as a pathogenic factor for ovarian dysgenesis: a case report. Front Endocrinol. 2020;11:540683. 10.3389/fendo.2020.540683 PMC7545356 33101191

[13] Eskenazi S , Bachelot A , Hugon‐Rodin J , Plu‐Bureau G , Gompel A , Catteau‐Jonard S , et al. Next generation sequencing should be proposed to every woman with “idiopathic” primary ovarian insufficiency. J Endocr Soc. 2021;5(7):bvab032. 34095689 10.1210/jendso/bvab032 PMC8169040

[14] Mei L , Huang Y , Wu X , He H , Ye R , Ma J , et al. Mutations in FIGLA associated with premature ovarian insufficiency in a Chinese population. Front Med. 2021;8:714306. 10.3389/fmed.2021.714306 PMC8585841 34778283

[15] Er E , Aşıkovalı S , Özışık H , Sağsak E , Gökşen D , Onay H , et al. Investigation of the molecular genetic causes of non‐syndromic primary ovarian insufficiency by next generation sequencing analysis. Arch Endocrinol Metab. 2023;68:e220475. 37988663 10.20945/2359-4292-2022-0475 PMC10916837

[16] Kawakami R , Hiraide T , Watanabe K , Miyamoto S , Hira K , Komatsu K , et al. RNA sequencing and target long‐read sequencing reveal an intronic transposon insertion causing aberrant splicing. J Hum Genet. 2024;69(2):91–99. 38102195 10.1038/s10038-023-01211-8

[17] Richards S , Aziz N , Bale S , Bick D , Das S , Gastier‐Foster J , et al. Standards and guidelines for the interpretation of sequence variants: a joint consensus recommendation of the American College of Medical Genetics and Genomics and the Association for Molecular Pathology. Genet Med. 2015;17(5):405–424. 25741868 10.1038/gim.2015.30 PMC4544753

[18] Moolhuijsen LME , Visser JA . Anti‐Müllerian hormone and ovarian reserve: update on assessing ovarian function. J Clin Endocrinol Metab. 2020;105(11):3361–3373. 32770239 10.1210/clinem/dgaa513 PMC7486884

[19] Broekmans FJ , Visser JA , Laven JS , Broer SL , Themmen AP , Fauser BC . Anti‐Mullerian hormone and ovarian dysfunction. Trends Endocrinol Metab. 2008;19(9):340–347. 18805020 10.1016/j.tem.2008.08.002

[20] Kuzmiak HA , Maquat LE . Applying nonsense‐mediated mRNA decay research to the clinic: progress and challenges. Trends Mol Med. 2006;12(7):306–316. 16782405 10.1016/j.molmed.2006.05.005

[21] Shekari S , Stankovic S , Gardner EJ , Hawkes G , Kentistou KA , Beaumont RN , et al. Penetrance of pathogenic genetic variants associated with premature ovarian insufficiency. Nat Med. 2023;29(7):1692–1699. 37349538 10.1038/s41591-023-02405-5

