# Supplementary material for: Homozygous FIGLA missense variant in two Japanese sisters with primary ovarian insufficiency: Case reports and literature review
Source: Reprod Med Biol. 2025 Feb 1;24(1):e12635. doi: 10.1002/rmb2.12635 (PMC11786018; doi:10.1002/rmb2.12635)
Supplement: Supplementary file 1 — Data S1. [file RMB2-24-e12635-s001.pdf]

Table S1. Assessment of consanguinity based on PI_HAT values calculated by PLINK

Family	IID1	IID2	Z0	Z1	Z2	PI_HAT
This family	Elder sister	Father	0.0048	0.9767	0.0185	0.5068
	Elder sister	Mother	0	0.9933	0.0067	0.5033
	Younger sister	Father	0.0097	0.9756	0.0147	0.5025
	Younger sister	Mother	0.0048	0.9803	0.0148	0.5050
	Elder sister	Younger sister	0.2944	0.4898	0.2158	0.4607
	Father	Mother	0.9531	0.0469	0	0.0234
Control*	Child	Father	0	1	0	0.5
	Child	Mother	0	1	0	0.5
	Father	Mother	0.7598	0.2402	0	0.1201

A total of 5,123 common exonic variants have been analyzed by PLINK 1.9 beta (https://www.cog-genomics.org/plink/1.9/).

IID1: Individual ID for first individual; IID2: Individual ID for second individual; Z0: P(IBD=0) (IBD: identity by descent); Z1: P(IBD=1); Z2: P(IBD=2); and PI_HAT: P(IBD=2)+0.5*P(IBD=1) (proportion IBD).

Theoretical PI_HAT values: 1.0 for the same individual or monozygotic twins; 0.5 for first-degree relatives; 0.25 for second-degree relatives, 0.125 for third-degree relatives, and 0 for unrelated individuals.

*For controls, PI_HAT values have been calculated for a family in which the parents are first cousins (third-degree relatives). The value of 0.1201 is similar to the expected value of 0.125.

Reference

Purcell S, Neale B, Todd-Brown K, Thomas L, Ferreira MAR, Bender B, et al. PLINK: A tool set for whole-genome association and population-based linkage analyses. Am J Hum Genet. 2007;81(3):559–575.

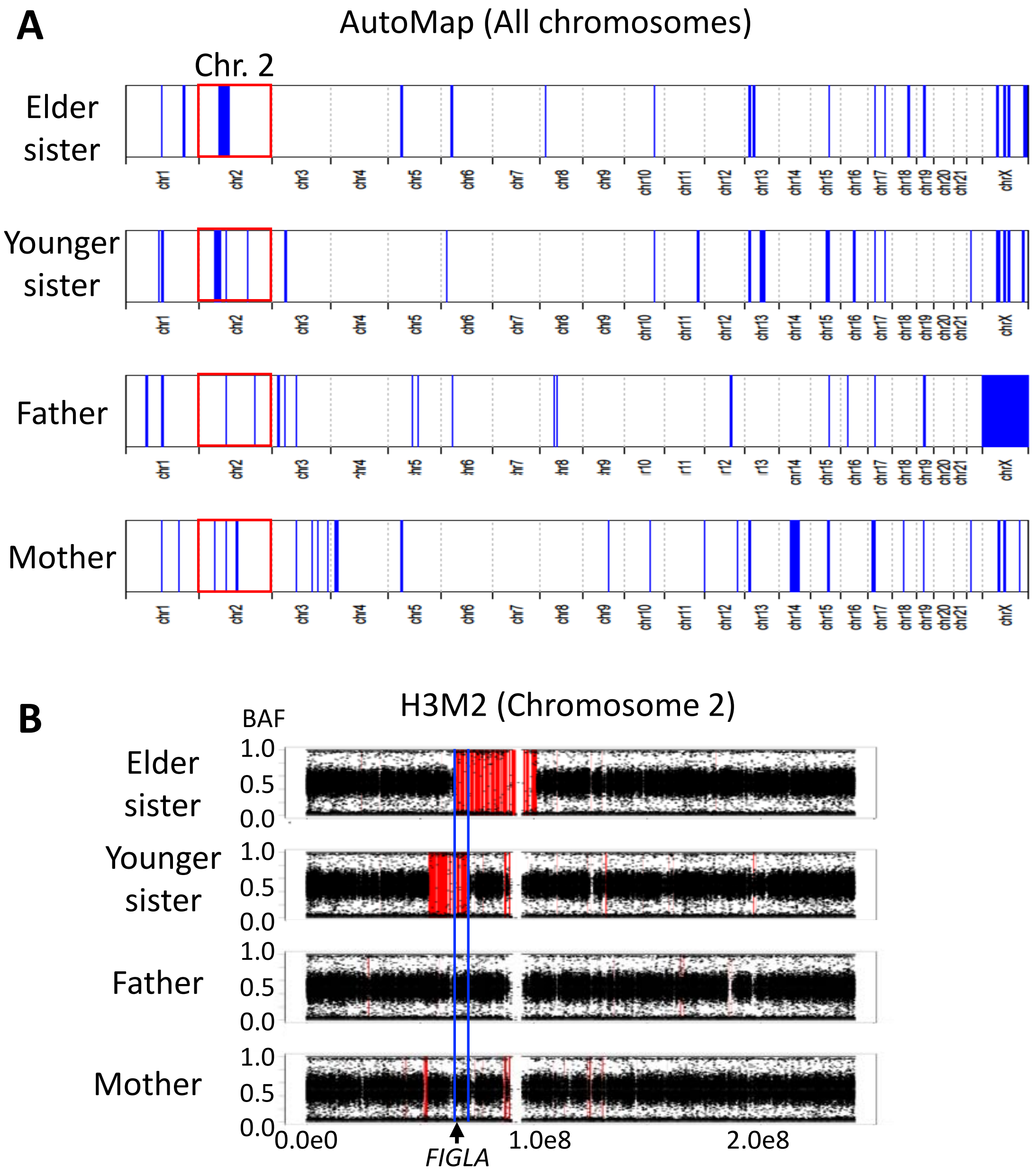

Supplementary Figure 1. Identification of ROH regions.

A. Automap analysis. The ROH regions are shown in blue. Chromosome 2 is indicated by red squares.

B. H3M2 analysis. The ROH regions are shown in red, and a region involving *FIGLA* is shared in common by the two sisters.
